# (*E*)-1,2-Bis(1-propyl-5,6-dimethyl-1*H*-benzimidazol-2-yl)ethene

**DOI:** 10.1107/S1600536810003405

**Published:** 2010-02-10

**Authors:** Robert T. Stibrany, Joseph A. Potenza

**Affiliations:** a Department of Chemistry and Chemical Biology, Rutgers, The State University of New Jersey, 610 Taylor Road, Piscataway, New Jersey 08854, USA

## Abstract

In the title compound, C_26_H_32_N_4_, the essentially planar (r.m.s. deviations of 0.0053 and 0.0242 Å) benzimidazole fragments are *trans* with respect to a central ethene fragment, and are canted in opposite directions by 2.78 (6) and 5.87 (6)° with respect to the ethene plane, giving the mol­ecule a propeller conformation. The terminal ethyl fragments of the pendant *n*-propyl groups protrude to either side of the benzimidazole planes. Overall, the mol­ecule exhibits a pseudo-center of symmetry at the mid-point of the ethene fragment. Both π–π stacking and typical C—H⋯π inter­actions are notably absent, as are inter­molecular hydrogen bonds. When viewed along the *a* axis, the structure appears as criss-crossed layers of mol­ecules with the planar fragments separated along the *c*-cell direction by the protruding ethyl groups.

## Related literature

For applications of bis­(imidazoles), bis­(benzimidazoles) and their complexes with metal ions, see: Knapp *et al.* (1990 ▶); Stibrany *et al.* (2002 ▶, 2003 ▶, 2004 ▶); Stibrany & Potenza (2008 ▶). The title compound was prepared from *rac*-1,2-bis­(1*H*-5,6-dimethyl­benzimidazol-2-yl)-1-hydroxy­ethane (Taffs *et al.*, 1961 ▶). Alkyl­ation was effected according to a reported method (Stibrany *et al.*, 2004 ▶). For related structures see: Stibrany *et al.* (2005 ▶); Stibrany & Potenza (2006*a* ▶,*b* ▶, 2009 ▶). For a description of the Cambridge Structural Database, see: Allen (2002 ▶).
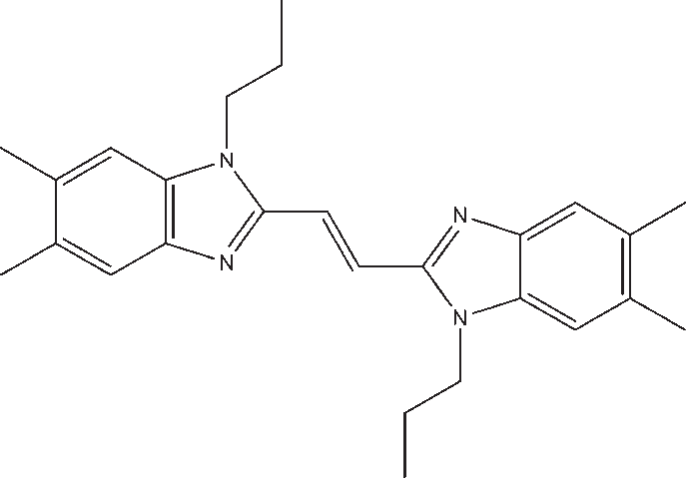


## Experimental

### 

#### Crystal data

C_26_H_32_N_4_
*M* *_r_* = 400.56
Monoclinic, 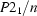

*a* = 12.7822 (15) Å
*b* = 10.4802 (12) Å
*c* = 16.5944 (19) Å
β = 100.284 (2)°
*V* = 2187.3 (4) Å^3^
*Z* = 4
Mo *K*α radiation
μ = 0.07 mm^−1^
*T* = 100 K
0.32 × 0.28 × 0.11 mm

#### Data collection

Bruker SMART CCD area-detector diffractometer
Absorption correction: multi-scan (*SADABS*; Blessing, 1995 ▶) *T*_min_ = 0.795, *T*_max_ = 1.00
20415 measured reflections
4324 independent reflections
3596 reflections with *I* > 2σ(*I*)
*R*_int_ = 0.041

#### Refinement

*R*[*F* ^2^ > 2σ(*F* ^2^)] = 0.057
*wR*(*F* ^2^) = 0.151
*S* = 1.00
4324 reflections
277 parameters
H-atom parameters constrained
Δρ_max_ = 0.34 e Å^−3^
Δρ_min_ = −0.18 e Å^−3^

### 

Data collection: *SMART* (Bruker, 2000 ▶); cell refinement: *SAINT-Plus* (Bruker, 2000 ▶); data reduction: *SAINT-Plus*; program(s) used to solve structure: *SHELXS97* (Sheldrick, 2008 ▶); program(s) used to refine structure: *SHELXL97* (Sheldrick, 2008 ▶); molecular graphics: *ORTEPIII* (Burnett & Johnson, 1996 ▶) and *ORTEP-32* (Farrugia, 1997 ▶); software used to prepare material for publication: *SHELXTL* (Sheldrick, 2008 ▶) and *PLATON* (Spek, 2009 ▶).

## Supplementary Material

Crystal structure: contains datablocks I, global. DOI: 10.1107/S1600536810003405/jh2128sup1.cif

Structure factors: contains datablocks I. DOI: 10.1107/S1600536810003405/jh2128Isup2.hkl

Additional supplementary materials:  crystallographic information; 3D view; checkCIF report

## Figures and Tables

**Fig. 1.**
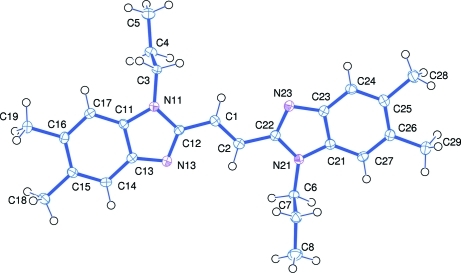
The molecular structure of the title compound showing the atom-numbering scheme. Displacement ellipsoids are shown at the 50% probability level. H atoms are shown as spheres of arbitrary radius

**Fig. 2.**
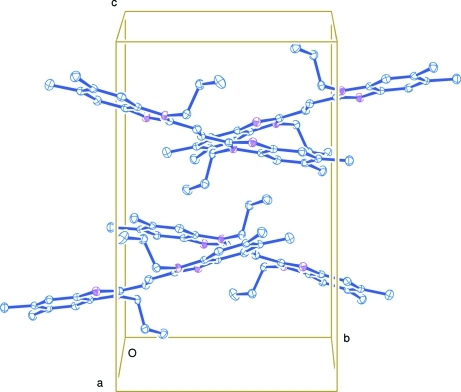
The structure viewed approximately along the a cell direction. H atoms have been omitted for clarity.

**Fig. 3.**
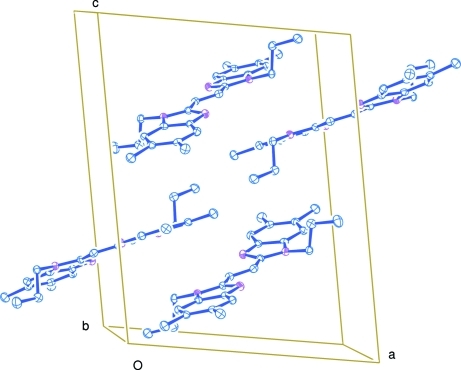
The structure viewed approximately along the b cell direction. H atoms have been omitted for clarity.

## supplementary crystallographic information

### Comment

The title compound (I) was prepared as part of our long-term interest in the chemistry of bis(imidazoles), bis(benzimidazoles), and their complexes with metal ions. These species have demonstrated their usefulness as proton sponges (Stibrany *et al.*, 2002), geometrically constraining ligands (Stibrany *et al.*, 2004), agents to study electron transfer (Knapp *et al.*, 1990), polymerization catalysts (Stibrany *et al.*, 2003), and in the formation of metal-organic copolymers (Stibrany & Potenza, 2008), The present structure (Fig. 1) contains a central, planar *trans *1,2-disubstituted ethene fragment linked at the 2 positions (C12 and C22) to 1-propyl, 5,6-dimethylbenzimidazole fragments. Excluding alkyl substituents, the structure can be viewed as three essentially planar fragments connected by two hinges, the C1—C12 and C2—C22 bonds. The benzimidazole fragments are canted in opposite directions by 2.78 (6)° (bzim 1) and 5.87 (6)°(bzim2) to give the molecule a slight propeller-like shape, while the ethyl fragments of the pendant *n*-propyl groups, which protrude above and below the planes of the benzimidazole fragments, help to ensure that the molecule has an approximate center of symmetry at the midpoint of the C1—C2 bond.

When viewed approximately along the a cell direction (Fig.2), the structure appears as layers of criss-crossed molecules with the planar fragments separated along the c cell direction by the protruding ethyl groups. A comparative view along the b cell direction (Fig. 3) shows the protruding ethyl groups in a different orientation and indicates clearly the lack of coplanarity of the benzimidazole fragments. These figures are consistent with the absence of π–π stacking and typical C—H···π interactions found by *Platon *(Spek, 2009). The absences noted above are consistent with the ethyl group conformations, which appear to prevent effective overlap of the π systems. The lack of intermolecular hydrogen bonds is also attributed to the *n*-propyl substituents, which prevent the formation of intermolecular *N*(imine)··· *H*–*N*(amine) hydrogen bonds.

### Experimental

Compound (I) was prepared from *rac*-1,2-bis(1*H*-5,6-dimethylbenzimidazol-2-yl)-1-hydroxyethane (Taffs *et al.*, 1961). Alkylation was effected according to a reported method (Stibrany *et al.*, 2004). Under Ar, NaH (6 molar equivalents) was added to amixture of *rac*-1,2-bis(1*H*-5,6-dimethylbenzimidazol-2-yl)-1-hydroxyethane in dry dimethyl sulfoxide (DMSO). After a reaction time of 10 minutes, *n*-propyl iodide (2 molar equivalents) was added dropwise. After an additional hour, the product was precipitated with water, collected by filtration, and dried in air. Crystals of (I) (m.p. 523 (soften) 538-539 K(melt)) were obtained by slow cooling of a hot DMSO solution of (I). *R*_f_ = 0.64 (ethyl acetate/silica). IR (KBr pellet, cm^-1^): 2967 (w), 2785 (w), 2700(w), 1582 (s), 1431 (m), 1367 (m), 1326 (w), 1004 (w), 768 (w), 669 (w), 649(w). Compound (I) is remarkably less soluble in a variety of solvents than analogous bis(benzimidazole)ethene compounds previously reported (Stibrany *et al.*, 2005; Stibrany & Potenza, 2006a,b; Stibrany & Potenza, 2009) which, in contrast to (I), were not substituted at the 5 and 6 positions.

### Refinement

Hydrogen atoms were positioned geometrically using a riding model, with C—H = 0.93 Å and *U*_iso_(H) = 1.2 *U*_eq _(C).

### Crystal data

**Table:**

C_26_H_32_N_4_	*F*(000) = 864
*M**_r_* = 400.56	*D*_x_ = 1.216 Mg m^−^^3^
Monoclinic, *P*2_1_/*n*	Mo *K*α radiation, λ = 0.71073 Å
Hall symbol: -P 2yn	Cell parameters from 953 reflections
*a* = 12.7822 (15) Å	θ = 2.3–25.6°
*b* = 10.4802 (12) Å	µ = 0.07 mm^−^^1^
*c* = 16.5944 (19) Å	*T* = 100 K
β = 100.284 (2)°	Plate, yellow
*V* = 2187.3 (4) Å^3^	0.32 × 0.28 × 0.11 mm
*Z* = 4	

### Data collection

**Table:**

Bruker SMART CCD area-detector diffractometer	4324 independent reflections
Radiation source: fine-focus sealed tube	3596 reflections with *I* > 2σ(*I*)
graphite	*R*_int_ = 0.041
φ and ω scans	θ_max_ = 26.1°, θ_min_ = 1.9°
Absorption correction: multi-scan (*SADABS*; Blessing, 1995)	*h* = −15→15
*T*_min_ = 0.795, *T*_max_ = 1.00	*k* = −12→12
20415 measured reflections	*l* = −20→20

### Refinement

**Table:**

Refinement on *F*^2^	Primary atom site location: structure-invariant direct methods
Least-squares matrix: full	Secondary atom site location: difference Fourier map
*R*[*F*^2^ > 2σ(*F*^2^)] = 0.057	Hydrogen site location: inferred from neighbouring sites
*wR*(*F*^2^) = 0.151	H-atom parameters constrained
*S* = 1.00	*w* = 1/[σ^2^(*F*_o_^2^) + (0.090*P*)^2^ + 1.071*P*] where *P* = (*F*_o_^2^ + 2*F*_c_^2^)/3
4324 reflections	(Δ/σ)_max_ < 0.001
277 parameters	Δρ_max_ = 0.34 e Å^−^^3^
0 restraints	Δρ_min_ = −0.18 e Å^−^^3^

### Special details

**Table:**

Geometry. All esds (except the esd in the dihedral angle between two l.s. planes) are estimated using the full covariance matrix. The cell esds are taken into account individually in the estimation of esds in distances, angles and torsion angles; correlations between esds in cell parameters are only used when they are defined by crystal symmetry. An approximate (isotropic) treatment of cell esds is used for estimating esds involving l.s. planes.
Refinement. Refinement of *F*^2^ against ALL reflections. The weighted *R*-factor wR and goodness of fit *S* are based on *F*^2^, conventional *R*-factors *R* are based on *F*, with *F* set to zero for negative *F*^2^. The threshold expression of *F*^2^ > σ(*F*^2^) is used only for calculating *R*-factors(gt) etc. and is not relevant to the choice of reflections for refinement. *R*-factors based on *F*^2^ are statistically about twice as large as those based on *F*, and *R*- factors based on ALL data will be even larger.

### Fractional atomic coordinates and isotropic or equivalent isotropic displacement parameters (Å^2^)

**Table:**

	*x*	*y*	*z*	*U*_iso_*/*U*_eq_	
N23	−0.03575 (12)	0.86314 (15)	0.21188 (9)	0.0185 (4)	
N13	0.04680 (12)	0.38211 (15)	0.29290 (9)	0.0184 (4)	
C13	0.12082 (14)	0.28848 (18)	0.32073 (10)	0.0162 (4)	
N11	0.20831 (12)	0.47168 (14)	0.32962 (9)	0.0164 (3)	
N21	−0.19800 (12)	0.77194 (14)	0.18947 (9)	0.0164 (3)	
C16	0.29931 (14)	0.13963 (18)	0.38184 (11)	0.0179 (4)	
C24	−0.10049 (14)	1.08026 (18)	0.15724 (11)	0.0188 (4)	
H24	−0.0327	1.1202	0.1677	0.023*	
C11	0.22163 (14)	0.34258 (17)	0.34411 (10)	0.0154 (4)	
C21	−0.21246 (14)	0.89625 (18)	0.16303 (10)	0.0168 (4)	
C26	−0.29142 (15)	1.08870 (18)	0.10388 (11)	0.0187 (4)	
C17	0.31163 (14)	0.27017 (18)	0.37496 (11)	0.0182 (4)	
H17	0.3792	0.3093	0.3907	0.022*	
C23	−0.11131 (14)	0.95179 (18)	0.17796 (10)	0.0170 (4)	
C15	0.19728 (14)	0.08225 (18)	0.35742 (10)	0.0175 (4)	
C14	0.10936 (14)	0.15596 (18)	0.32759 (11)	0.0179 (4)	
H14	0.0416	0.1172	0.3118	0.021*	
C25	−0.18941 (15)	1.14824 (18)	0.12140 (11)	0.0191 (4)	
C12	0.10150 (14)	0.49004 (17)	0.29953 (11)	0.0164 (4)	
C22	−0.09024 (14)	0.75650 (18)	0.21683 (11)	0.0167 (4)	
C27	−0.30311 (14)	0.96279 (18)	0.12593 (11)	0.0180 (4)	
H27	−0.3709	0.9227	0.1161	0.022*	
C2	−0.04547 (15)	0.63331 (18)	0.24519 (11)	0.0186 (4)	
H2	−0.0918	0.5619	0.2418	0.022*	
C1	0.05794 (14)	0.61551 (18)	0.27588 (11)	0.0178 (4)	
H1	0.1042	0.6872	0.2824	0.021*	
C19	0.39431 (15)	0.05820 (19)	0.41661 (12)	0.0221 (4)	
H19A	0.4580	0.1119	0.4284	0.033*	
H19B	0.4045	−0.0075	0.3767	0.033*	
H19C	0.3821	0.0174	0.4673	0.033*	
C18	0.18662 (16)	−0.06113 (18)	0.36216 (12)	0.0228 (4)	
H18A	0.1118	−0.0852	0.3457	0.034*	
H18B	0.2125	−0.0895	0.4185	0.034*	
H18C	0.2288	−0.1016	0.3254	0.034*	
C28	−0.17841 (16)	1.28882 (19)	0.10451 (13)	0.0251 (5)	
H28A	−0.1030	1.3123	0.1150	0.038*	
H28B	−0.2161	1.3385	0.1404	0.038*	
H28C	−0.2090	1.3068	0.0472	0.038*	
C29	−0.38687 (16)	1.1613 (2)	0.06029 (12)	0.0250 (5)	
H29A	−0.4496	1.1060	0.0533	0.037*	
H29B	−0.3741	1.1888	0.0065	0.037*	
H29C	−0.3989	1.2362	0.0928	0.037*	
C6	−0.28135 (14)	0.67505 (18)	0.17675 (11)	0.0175 (4)	
H6A	−0.3499	0.7144	0.1829	0.021*	
H6B	−0.2645	0.6078	0.2190	0.021*	
C3	0.29247 (14)	0.56624 (17)	0.35368 (11)	0.0167 (4)	
H3A	0.2768	0.6433	0.3192	0.020*	
H3B	0.3611	0.5308	0.3443	0.020*	
C4	0.30192 (15)	0.60307 (19)	0.44332 (11)	0.0210 (4)	
H4A	0.2370	0.6497	0.4509	0.025*	
H4B	0.3067	0.5246	0.4770	0.025*	
C5	0.39858 (16)	0.6861 (2)	0.47298 (12)	0.0248 (4)	
H5A	0.4634	0.6375	0.4703	0.037*	
H5B	0.3986	0.7124	0.5297	0.037*	
H5C	0.3961	0.7618	0.4381	0.037*	
C7	−0.29199 (15)	0.61516 (19)	0.09218 (12)	0.0222 (4)	
H7A	−0.3002	0.6833	0.0502	0.027*	
H7B	−0.2265	0.5669	0.0884	0.027*	
C8	−0.38697 (17)	0.5262 (2)	0.07528 (14)	0.0312 (5)	
H8A	−0.3838	0.4657	0.1207	0.047*	
H8B	−0.3858	0.4793	0.0244	0.047*	
H8C	−0.4527	0.5761	0.0699	0.047*	

### Atomic displacement parameters (Å^2^)

**Table:**

	*U*^11^	*U*^22^	*U*^33^	*U*^12^	*U*^13^	*U*^23^
N23	0.0164 (8)	0.0184 (8)	0.0205 (8)	0.0018 (6)	0.0026 (6)	0.0001 (6)
N13	0.0171 (8)	0.0177 (8)	0.0194 (8)	0.0012 (6)	0.0008 (6)	0.0012 (6)
C13	0.0158 (9)	0.0196 (10)	0.0134 (8)	0.0000 (7)	0.0030 (7)	−0.0004 (7)
N11	0.0164 (8)	0.0155 (8)	0.0169 (7)	−0.0006 (6)	0.0017 (6)	−0.0001 (6)
N21	0.0160 (8)	0.0153 (8)	0.0179 (7)	0.0006 (6)	0.0028 (6)	0.0006 (6)
C16	0.0191 (9)	0.0193 (10)	0.0150 (8)	0.0041 (7)	0.0023 (7)	0.0000 (7)
C24	0.0178 (9)	0.0186 (10)	0.0207 (9)	−0.0016 (7)	0.0053 (7)	−0.0018 (7)
C11	0.0188 (9)	0.0137 (9)	0.0138 (8)	−0.0003 (7)	0.0035 (7)	−0.0004 (7)
C21	0.0200 (9)	0.0172 (9)	0.0131 (8)	0.0006 (7)	0.0027 (7)	−0.0020 (7)
C26	0.0204 (10)	0.0193 (10)	0.0163 (9)	0.0032 (8)	0.0032 (7)	−0.0013 (7)
C17	0.0153 (9)	0.0203 (10)	0.0184 (9)	−0.0006 (7)	0.0012 (7)	−0.0006 (7)
C23	0.0175 (9)	0.0195 (10)	0.0144 (8)	0.0020 (7)	0.0038 (7)	−0.0012 (7)
C15	0.0226 (10)	0.0161 (9)	0.0137 (8)	−0.0021 (7)	0.0028 (7)	0.0005 (7)
C14	0.0171 (9)	0.0190 (10)	0.0173 (9)	−0.0043 (7)	0.0025 (7)	−0.0022 (7)
C25	0.0247 (10)	0.0178 (10)	0.0160 (9)	0.0014 (8)	0.0070 (7)	0.0002 (7)
C12	0.0160 (9)	0.0184 (9)	0.0144 (8)	−0.0002 (7)	0.0015 (7)	−0.0003 (7)
C22	0.0169 (9)	0.0181 (9)	0.0149 (8)	0.0013 (7)	0.0025 (7)	−0.0014 (7)
C27	0.0173 (9)	0.0203 (10)	0.0160 (9)	−0.0011 (7)	0.0016 (7)	−0.0022 (7)
C2	0.0196 (10)	0.0186 (10)	0.0183 (9)	−0.0011 (7)	0.0056 (7)	−0.0003 (7)
C1	0.0200 (10)	0.0158 (9)	0.0179 (9)	0.0002 (7)	0.0041 (7)	−0.0003 (7)
C19	0.0214 (10)	0.0190 (10)	0.0244 (10)	0.0032 (8)	0.0004 (8)	0.0024 (8)
C18	0.0263 (10)	0.0168 (10)	0.0242 (10)	−0.0014 (8)	0.0014 (8)	0.0001 (8)
C28	0.0267 (11)	0.0204 (11)	0.0284 (10)	0.0008 (8)	0.0056 (8)	0.0039 (8)
C29	0.0247 (11)	0.0237 (11)	0.0252 (10)	0.0037 (8)	0.0010 (8)	0.0033 (8)
C6	0.0154 (9)	0.0172 (9)	0.0198 (9)	−0.0013 (7)	0.0029 (7)	0.0016 (7)
C3	0.0144 (9)	0.0140 (9)	0.0216 (9)	−0.0023 (7)	0.0030 (7)	0.0014 (7)
C4	0.0226 (10)	0.0190 (10)	0.0221 (10)	−0.0033 (8)	0.0056 (8)	−0.0007 (8)
C5	0.0255 (10)	0.0245 (11)	0.0236 (10)	−0.0039 (9)	0.0022 (8)	−0.0044 (8)
C7	0.0245 (10)	0.0189 (10)	0.0226 (10)	0.0014 (8)	0.0028 (8)	−0.0011 (8)
C8	0.0253 (11)	0.0297 (12)	0.0370 (12)	0.0001 (9)	0.0013 (9)	−0.0121 (10)

### Geometric parameters (Å, °)

**Table:**

N23—C22	1.327 (2)	C2—H2	0.9500
N23—C23	1.385 (2)	C1—H1	0.9500
N13—C12	1.324 (2)	C19—H19A	0.9800
N13—C13	1.384 (2)	C19—H19B	0.9800
C13—C11	1.398 (2)	C19—H19C	0.9800
C13—C14	1.403 (3)	C18—H18A	0.9800
N11—C11	1.379 (2)	C18—H18B	0.9800
N11—C12	1.381 (2)	C18—H18C	0.9800
N11—C3	1.465 (2)	C28—H28A	0.9800
N21—C21	1.377 (2)	C28—H28B	0.9800
N21—C22	1.381 (2)	C28—H28C	0.9800
N21—C6	1.460 (2)	C29—H29A	0.9800
C16—C17	1.384 (3)	C29—H29B	0.9800
C16—C15	1.428 (3)	C29—H29C	0.9800
C16—C19	1.512 (3)	C6—C7	1.521 (3)
C24—C25	1.383 (3)	C6—H6A	0.9900
C24—C23	1.403 (3)	C6—H6B	0.9900
C24—H24	0.9500	C3—C4	1.521 (3)
C11—C17	1.397 (3)	C3—H3A	0.9900
C21—C27	1.397 (3)	C3—H3B	0.9900
C21—C23	1.399 (3)	C4—C5	1.519 (3)
C26—C27	1.385 (3)	C4—H4A	0.9900
C26—C25	1.428 (3)	C4—H4B	0.9900
C26—C29	1.508 (3)	C5—H5A	0.9800
C17—H17	0.9500	C5—H5B	0.9800
C15—C14	1.381 (3)	C5—H5C	0.9800
C15—C18	1.512 (3)	C7—C8	1.517 (3)
C14—H14	0.9500	C7—H7A	0.9900
C25—C28	1.511 (3)	C7—H7B	0.9900
C12—C1	1.454 (3)	C8—H8A	0.9800
C22—C2	1.456 (3)	C8—H8B	0.9800
C27—H27	0.9500	C8—H8C	0.9800
C2—C1	1.342 (3)		
			
C22—N23—C23	104.73 (15)	H19A—C19—H19B	109.5
C12—N13—C13	104.98 (15)	C16—C19—H19C	109.5
N13—C13—C11	110.28 (16)	H19A—C19—H19C	109.5
N13—C13—C14	130.83 (17)	H19B—C19—H19C	109.5
C11—C13—C14	118.90 (17)	C15—C18—H18A	109.5
C11—N11—C12	106.44 (15)	C15—C18—H18B	109.5
C11—N11—C3	123.67 (15)	H18A—C18—H18B	109.5
C12—N11—C3	129.41 (15)	C15—C18—H18C	109.5
C21—N21—C22	106.56 (15)	H18A—C18—H18C	109.5
C21—N21—C6	124.01 (15)	H18B—C18—H18C	109.5
C22—N21—C6	128.73 (15)	C25—C28—H28A	109.5
C17—C16—C15	120.30 (17)	C25—C28—H28B	109.5
C17—C16—C19	119.60 (17)	H28A—C28—H28B	109.5
C15—C16—C19	120.09 (17)	C25—C28—H28C	109.5
C25—C24—C23	119.38 (17)	H28A—C28—H28C	109.5
C25—C24—H24	120.3	H28B—C28—H28C	109.5
C23—C24—H24	120.3	C26—C29—H29A	109.5
N11—C11—C17	131.66 (17)	C26—C29—H29B	109.5
N11—C11—C13	105.69 (16)	H29A—C29—H29B	109.5
C17—C11—C13	122.65 (17)	C26—C29—H29C	109.5
N21—C21—C27	131.78 (17)	H29A—C29—H29C	109.5
N21—C21—C23	105.66 (15)	H29B—C29—H29C	109.5
C27—C21—C23	122.53 (17)	N21—C6—C7	111.30 (15)
C27—C26—C25	120.02 (17)	N21—C6—H6A	109.4
C27—C26—C29	119.42 (17)	C7—C6—H6A	109.4
C25—C26—C29	120.55 (17)	N21—C6—H6B	109.4
C16—C17—C11	117.96 (17)	C7—C6—H6B	109.4
C16—C17—H17	121.0	H6A—C6—H6B	108.0
C11—C17—H17	121.0	N11—C3—C4	111.38 (14)
N23—C23—C21	110.38 (16)	N11—C3—H3A	109.4
N23—C23—C24	130.64 (17)	C4—C3—H3A	109.4
C21—C23—C24	118.97 (17)	N11—C3—H3B	109.4
C14—C15—C16	120.66 (17)	C4—C3—H3B	109.4
C14—C15—C18	119.97 (17)	H3A—C3—H3B	108.0
C16—C15—C18	119.34 (17)	C5—C4—C3	112.34 (16)
C15—C14—C13	119.53 (17)	C5—C4—H4A	109.1
C15—C14—H14	120.2	C3—C4—H4A	109.1
C13—C14—H14	120.2	C5—C4—H4B	109.1
C24—C25—C26	120.87 (17)	C3—C4—H4B	109.1
C24—C25—C28	119.06 (17)	H4A—C4—H4B	107.9
C26—C25—C28	120.01 (17)	C4—C5—H5A	109.5
N13—C12—N11	112.60 (16)	C4—C5—H5B	109.5
N13—C12—C1	125.38 (16)	H5A—C5—H5B	109.5
N11—C12—C1	121.99 (16)	C4—C5—H5C	109.5
N23—C22—N21	112.63 (16)	H5A—C5—H5C	109.5
N23—C22—C2	125.90 (16)	H5B—C5—H5C	109.5
N21—C22—C2	121.45 (16)	C8—C7—C6	111.16 (16)
C26—C27—C21	118.16 (17)	C8—C7—H7A	109.4
C26—C27—H27	120.9	C6—C7—H7A	109.4
C21—C27—H27	120.9	C8—C7—H7B	109.4
C1—C2—C22	123.34 (18)	C6—C7—H7B	109.4
C1—C2—H2	118.3	H7A—C7—H7B	108.0
C22—C2—H2	118.3	C7—C8—H8A	109.5
C2—C1—C12	122.13 (18)	C7—C8—H8B	109.5
C2—C1—H1	118.9	H8A—C8—H8B	109.5
C12—C1—H1	118.9	C7—C8—H8C	109.5
C16—C19—H19A	109.5	H8A—C8—H8C	109.5
C16—C19—H19B	109.5	H8B—C8—H8C	109.5
			
C12—N13—C13—C11	−0.16 (19)	C23—C24—C25—C26	−1.2 (3)
C12—N13—C13—C14	−179.65 (18)	C23—C24—C25—C28	175.89 (17)
C12—N11—C11—C17	−179.41 (18)	C27—C26—C25—C24	2.7 (3)
C3—N11—C11—C17	−6.7 (3)	C29—C26—C25—C24	−176.39 (17)
C12—N11—C11—C13	0.71 (18)	C27—C26—C25—C28	−174.33 (17)
C3—N11—C11—C13	173.39 (15)	C29—C26—C25—C28	6.6 (3)
N13—C13—C11—N11	−0.36 (19)	C13—N13—C12—N11	0.6 (2)
C14—C13—C11—N11	179.20 (15)	C13—N13—C12—C1	178.64 (17)
N13—C13—C11—C17	179.75 (16)	C11—N11—C12—N13	−0.9 (2)
C14—C13—C11—C17	−0.7 (3)	C3—N11—C12—N13	−172.99 (16)
C22—N21—C21—C27	176.43 (18)	C11—N11—C12—C1	−178.96 (16)
C6—N21—C21—C27	5.3 (3)	C3—N11—C12—C1	8.9 (3)
C22—N21—C21—C23	−1.54 (18)	C23—N23—C22—N21	−1.3 (2)
C6—N21—C21—C23	−172.65 (15)	C23—N23—C22—C2	176.97 (16)
C15—C16—C17—C11	0.4 (3)	C21—N21—C22—N23	1.9 (2)
C19—C16—C17—C11	−178.72 (16)	C6—N21—C22—N23	172.42 (16)
N11—C11—C17—C16	−179.49 (18)	C21—N21—C22—C2	−176.52 (16)
C13—C11—C17—C16	0.4 (3)	C6—N21—C22—C2	−6.0 (3)
C22—N23—C23—C21	0.30 (19)	C25—C26—C27—C21	−1.9 (3)
C22—N23—C23—C24	−178.94 (18)	C29—C26—C27—C21	177.25 (16)
N21—C21—C23—N23	0.80 (19)	N21—C21—C27—C26	−178.11 (18)
C27—C21—C23—N23	−177.40 (15)	C23—C21—C27—C26	−0.4 (3)
N21—C21—C23—C24	−179.86 (15)	N23—C22—C2—C1	5.1 (3)
C27—C21—C23—C24	1.9 (3)	N21—C22—C2—C1	−176.72 (17)
C25—C24—C23—N23	178.11 (17)	C22—C2—C1—C12	−176.52 (16)
C25—C24—C23—C21	−1.1 (3)	N13—C12—C1—C2	0.9 (3)
C17—C16—C15—C14	−0.8 (3)	N11—C12—C1—C2	178.73 (16)
C19—C16—C15—C14	178.29 (16)	C21—N21—C6—C7	83.2 (2)
C17—C16—C15—C18	177.44 (17)	C22—N21—C6—C7	−85.9 (2)
C19—C16—C15—C18	−3.5 (2)	C11—N11—C3—C4	−81.9 (2)
C16—C15—C14—C13	0.5 (3)	C12—N11—C3—C4	89.0 (2)
C18—C15—C14—C13	−177.76 (16)	N11—C3—C4—C5	171.72 (15)
N13—C13—C14—C15	179.71 (17)	N21—C6—C7—C8	−173.22 (16)
C11—C13—C14—C15	0.3 (2)		
